# Natural Killer Cells: Angels and Devils for Immunotherapy

**DOI:** 10.3390/ijms18091868

**Published:** 2017-08-29

**Authors:** Beatriz Martín-Antonio, Guillermo Suñe, Lorena Perez-Amill, Maria Castella, Alvaro Urbano-Ispizua

**Affiliations:** 1 Department of Hematology, Hospital Clinic, Institut d’Investigacions Biomèdiques August Pi i Sunyer (IDIBAPS), 08036 Barcelona, Spain; gsune@carrerasresearch.org (G.S.); loperez@clinic.ub.es (L.P.-A.); mcastella@clinic.cat (M.C.); aurbano@clinic.ub.es (A.U.-I.); 2 Josep Carreras Leukaemia Research Institute, 08036 Barcelona, Spain

**Keywords:** natural killer (NK), immunotherapy, tumor cell survival mechanisms, inflammation

## Abstract

In recent years, the relevance of the immune system to fight cancer has led to the development of immunotherapy, including the adoptive cell transfer of immune cells, such as natural killer (NK) cells and chimeric antigen receptors (CAR)-modified T cells. The discovery of donor NK cells’ anti-tumor activity in acute myeloid leukemia patients receiving allogeneic stem cell transplantation (allo-SCT) was the trigger to conduct many clinical trials infusing NK cells. Surprisingly, many of these studies did not obtain optimal results, suggesting that many different NK cell parameters combined with the best clinical protocol need to be optimized. Various parameters including the high array of activating receptors that NK cells have, the source of NK cells selected to treat patients, different cytotoxic mechanisms that NK cells activate depending on the target cell and tumor cell survival mechanisms need to be considered before choosing the best immunotherapeutic strategy using NK cells. In this review, we will discuss these parameters to help improve current strategies using NK cells in cancer therapy. Moreover, the chimeric antigen receptor (CAR) modification, which has revolutionized the concept of immunotherapy, will be discussed in the context of NK cells. Lastly, the dark side of NK cells and their involvement in inflammation will also be discussed.

## 1. Natural Killer (NK) Cell Modulation Activity

Natural killer (NK) cells are cells of the innate immune system with high anti-tumor, antiviral and antimicrobial activity. In healthy individuals, 90% of NK cells in peripheral blood (PB) are mature and cytotoxic, and characterized by the expression of CD16^bright^ and CD56^dim^. The remaining 10% of NK cells represent the immature subset of NK cells that are cytokine producers, and express CD16^dim^ or CD16^−^, CD56^bright^ and CD25^+^ [1]. The activity of NK cells is modulated by an array of inhibitory and activating receptors, which are fundamental in controlling NK cell activity. Whereas inhibitory Killer-cell immunoglobulin-like (KIR) receptors inhibit NK cell cytotoxicity by interacting with the Human Leukocyte Antigen (HLA)-I in human cells, activating receptors activate NK cells by interacting with their ligands in target cells induced upon tumor transformation, viral infection and cell stress. In physiological conditions, inhibitory KIR-HLA-I interaction holds NK cell cytotoxicity against normal cells in the body. However, when a KIR-HLA-I mismatch or HLA-I down-regulation occurs after viral infection or in some tumor cells, inhibitory KIRs cannot interact with their ligands activating NK cell cytotoxic function [2]. This finding observed by Ruggeri et al. in 2002 in acute myeloblastic leukemia (AML) patients after allogeneic stem cell transplantation (allo-SCT), allowed using the inhibitory KIR (donor)-HLA-I (patient) mismatch as a prognostic factor in AML patients receiving an allo-SCT [3]. This “missing self” recognition leads to allo-reactivity after allo-SCT, and consequently, NK cells lyse leukemia blasts, recipient dendritic cells (DCs) and recipient T cells, which translates into a reduction of relapse, prevention of Graft vs. Host Disease (GVHD), and prevention of graft rejection, respectively. The “missing self” recognition led to the proposal of the “missing ligand model” as a powerful algorithm to predict a potent anti-leukemia effect and, consequently, a favorable outcome after allo-SCT [3,4]. However, in non-myeloid malignancies, such as acute lymphoblastic leukemia (ALL), the GVHD reduction and prevention of graft rejection was not always accompanied by an increase of the graft vs. leukemia effect observed in AML [5]. These conflicting results led to applying the “missing ligand model” mostly in AML [6], and in non-myeloid malignancies to use this model just for NK-mediated killing of DCs and T cells to predict a reduced GVHD and reduced graft rejection, respectively. The graft vs. leukemia effect occurring mostly in AML could be mediated by HLA down-regulation, which does not occur in other malignancies such as ALL. To support this, clinical studies in ALL confirmed that low HLA-I expression levels in blasts conferred a beneficial effect mediated by allo-reactive NK cells [7].

However, NK cells have a high variety of activating receptors, which also modulate their cytotoxic activity. These receptors will play a fundamental role in the recognition of other types of tumor cells which do not down-regulate HLA-I. Some of these receptors are expressed only at given stages of differentiation or by specific NK cell subsets [8]. Surprisingly, this high number of activating receptors in NK cells is responsible for the existence of 6000–30,000 different phenotypic NK populations in each healthy individual, which provide flexibility to respond to pathogens and tumor cells [9]. Activating receptors include different families such as the: (i) activating KIRs which interact with TYRO protein tyrosine kinase binding protein (DAP-12); (ii) C-type lectin-like receptors, including the activating CD94/NKG2C and NKG2D. NKP80, included in this family, exerts an autonomous control of NK cells against excessive inflammatory response causing self-NK cells mediated cytolysis [10,11,12]; (iii) natural cytotoxicity receptors (NCR) which include NKP30, NKP44 and NKP46 and interact with ligands overexpressed on tumor cells and viral infected cells; and (iv) signaling lymphocyte activating molecule (SLAM) family of receptors which include SLAMF1, 2B4, NTB-A, CD48, CD84, Ly9, and CRACC. They transmit activating signals to mediate NK cytotoxicity. Moreover, NK cells express CD16, which is the Fc receptor that binds Ig-G mediating antibody dependent cellular cytotoxicity (ADCC).

## 2. NK Cell Classic Cytotoxicity Mechanisms

Upon recognition, NK cells eliminate target cells rapidly (within 30–60 min) by different mechanisms. The two classic NK cell cytotoxic mechanisms include the death receptor pathway and the granule dependent pathway. The death receptor pathway is activated by the tumor necrosis factor (TNF)-related apoptosis inducing ligand family (TRAIL) and by Fas-Ligand (FASL) (CD95L) which are expressed on NK cells [13], and interact with their ligands in target cells. Interaction of FASL with FAS and TRAIL with TRAIL receptors allows the formation of a death-inducing signaling complex that includes Fas-associated death domain protein (FADD), caspase-8, and caspase-10 [14]. Activation of caspase-8 results either in direct activation of the other caspases or in the proteolysis of Bid, with release of cytochrome C and subsequent caspase activation. Even if it were believed that these receptors only activate apoptosis, new studies have revealed that other types of non-apoptotic inflammatory types of cell death can be activated [15,16]. The granule dependent pathway is initiated after NK cells adhere to the target cell, with subsequent delivery of cytotoxic granules containing perforin and proteases called Granzymes toward the bound target cell [17]. In humans, there are five different types of Granzymes (A, B, H, K and M) and each one will activate different cell death pathways, either apoptotic or non-apoptotic. Granzymes A and B are the most studied ones. Whereas Granzyme B activates apoptosis through activation of caspases or release of cytochrome C from the mitochondria, apparently, Granzyme A activates non-apoptotic cell death [18]; however, there are conflicting results regarding the cytotoxic role of Granzyme A [19]. The other Granzymes have been less studied, although it has been shown that Granzyme K is released by CD56bright cells mediating non-apoptotic tumor cell death [20]. Granzyme M shows anti-tumor properties after adoptive NK cell transfer [21]. Granzyme H helps Granzyme B kill adenovirus-infected cells [22]; and Granulysin, which is another NK cytotoxic molecule released in the cytotoxic granules, activates Endoplasmatic Reticulum stress leading to cell death [23]. All these different cytotoxicity mechanisms that NK cells show, enable them to eliminate different types of tumor cells with their intrinsic characteristics.

## 3. Immunotherapy Strategies Using NK Cells

As previously mentioned, the anti-tumor NK cell activity described after allo-SCT added to the finding that unlike donor T cells, NK cells do not induce GVHD, led to the development of many clinical trials infusing NK cells in patients receiving an SCT. We have reviewed published studies infusing NK cells as an immunotherapy option. Results are not optimal; they are summarized in Table 1. In most of these studies, NK cells were activated in vitro and/or in vivo with Interleukin (IL)2, and administered after immunosuppressive treatment based on fludarabine and cyclophosphamide. Patient disease, disease status, number of NK cells infused and number of NK cell infusions differed from one study to another. All published studies agree that NK cell infusion is a safe and well-tolerated procedure and does not associate to GVHD. Whereas, for myeloid malignancies, most studies used NK cells from haploidentical donors considering the KIR-HLA-I mismatch, in non-myeloid malignancies, NK cells were either autologous or allogeneic and expanded in vitro.

In myeloid malignancies, haploidentical NK cells with KIR-HLA-I mismatch have been used either as a consolidation therapy [24,25] or in high risk and refractory patients [26,27]. As a consolidation therapy, in both children with AML and elderly AML patients, NK cells showed a clear benefit. After a median follow up of four years, pediatric patients remain in remission, and elderly patients with high risk AML showed prolonged disease free survival (DFS) after NK cell infusion [24,25,28]. These studies concluded that NK cells could be a promising consolidation therapy strategy in patients who are not candidates for receiving an allo-SCT. Another novel consolidation therapy strategy in AML consisted on the infusion of NK cells derived from hematopoietic progenitor cells (HPC) obtained from a cord blood (CB) unit. This technique allowed obtaining enough number of NK cells which were well tolerated. Unlike other procedures, this study did not administrate IL2 into patients, and responses were detected, suggesting that this technique allows obtaining effective NK cells which might be used off-the-shelf when required [29]. 

In refractory patients, results are not good. However, it was noticed that infusion of haploidentical NK cells combined with IL2 diphtheria toxin fusion protein (IL2DT) to deplete host T-reg cells led to higher NK cell expansion, improved complete remission rate and disease-free survival with no increased toxicity in AML patients [26]. Lee et al. also showed durable responses associated with CD56+ cells delivered in myeloid malignancies [27]. Another study in AML and MDS found resolution of dysplastic features in 50% of myelodisplastic sindrome (MDS) patients, and 16% of complete remission (CR) in AML; however, they could not find NK cells in peripheral blood (PB) [30]. In pediatric refractory AML patients who received or did not receive an allo-SCT previously, responses higher than 50% were detected after NK infusion, and combined with allo-SCT afterwards, 27% and 36% of DFS were achieved, respectively, at six years [31]. Another approach tested in high risk AML patients, consisted in the infusion of haploidentical NK cells previously primed with tumor cell lysates. IL2 was not administrated to patients, and still NK cells seemed to exert anti-leukemia effect in 57% of the patients. However, at 2 years 85.7% patients died [32]. Studies in myeloid malignancies indicated that better responses were achieved when infusing NK cells in patients in remission suggesting that NK cells cannot overcome large tumor burdens. Therefore, more recent approaches aim at expanding NK cells in vitro to obtain a high number of NK for large tumor mass. 

**Figure 1:**
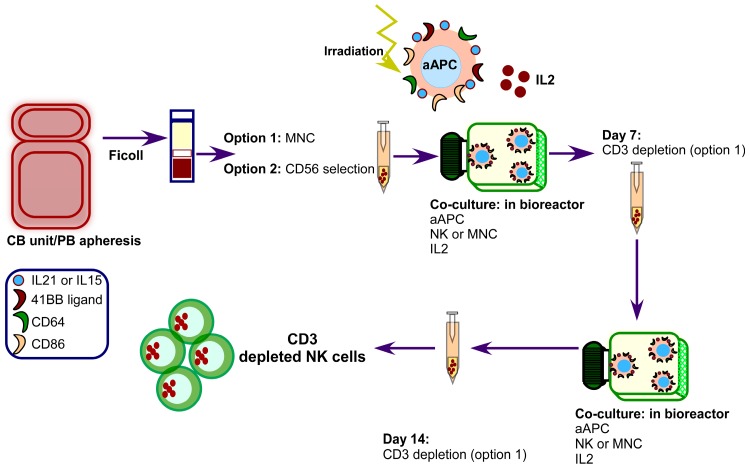
Clinical expansion of Natural Killer (NK) cells from apheresis products or cord blood (CB) units. Activated NK cells can be generated starting either from mononuclear cells (MNC) or with magnetically selected NK cells. First, either CB or apheresis products are ficolled to get the MNC, and then they are either added directly to a bio-reactor or subjected to magnetic CD56+ selection. These CD56+ cells will be added to the bio-reactor. Then, they are expanded in vitro for seven days with artificial antigen presenting cells (aAPCs). IL2 is added exogenously every other day. aAPCs are K562-based aAPCs expressing 41BB ligand, CD64, CD86 and either membrane bound IL21 or IL15. They are co-cultured in a 2:1 aAPC:MNC or NK cells ratio. On Day 7, fresh aAPCs are added again in the same ratio and co-cultured in the same conditions for an additional seven days. On Day 7 and Day 14, cells are CD3-depleted, only in case the expansion was started with MNC. Expansion can be continued for a total of 4 weeks repeating the same procedure. PB: peripheral blood.

In non-myeloid malignancies, both haploidentical NK cells and NK cells expanded in vitro from other sources have been used. Infusion of haploidentical NK cells in multiple myeloma (MM) relapsed patients, before an autologous-SCT (auto-SCT), obtained 50% of complete or near complete responses [33]. Yang et al. expanded allogeneic NK cells in vitro allowing infusion of repetitive administrations of NK cells in advanced lymphoma and advanced solid tumors. They found that activated and expanded NK cells are also safe obtaining 47.1% of stable disease. Interestingly, they observed that T-reg cells and myeloid-derived suppressor cells were reduced after NK administration [34]. In relapsed MM, different protocols infusing in vitro expanded NK cells with K562 artificial Antigen Presenting Cells (aAPCs) have been tested. For instance, Leivas et al. combining autologous expanded NK cells with anti-MM drugs (Lenalidomide/Bortezomib) showed 80% of disease stabilization and that the combination of NK cells with Lenalidomide was the best one. They did not administrate IL2 into the patients [35]. Szmania et al. infused either autologous or haploidentical expanded NK cells combined with Bortezomib-Dexamethasone based anti-MM treatment, and IL2 in vivo administration, obtaining much lower responses (28% PR) [36]. Shah et al. [37] infused expanded NK cells derived from a CB unit (CB-NK) and combined them with Lenalidomide before auto-SCT. Eighty-three percent of patients showed very good partial responses; at 21 months, 33% of patients relapsed. These three studies showed that NK cell in vitro expansion using aAPC K562-based system is an efficient technique to obtain a high number of activated NK cells, which are safe for patients. The NK cells in vitro expansion technique with aAPC K562-based system can be visualized in Figure 1.

In the context of non-hematological malignancies, haploidentical NK cells have been used with no optimal results. In pediatric patients with refractory solid tumors, haploidentical NK cells were infused after haplo-SCT. No toxic effects were observed, and 66% of the patients showed a clinical response. However, all patients had died after 310 days [38]. In adults with recurrent ovarian and breast cancer, the beneficial effect of NK cells could not be differentiated from the chemotherapy regimen [39]. Another approach in hepatocellular carcinoma combined allogeneic in vitro expanded NK cells showing KIR-HLA-I mismatch with cryosurgery. Beneficial effects were detected in terms of enhanced immune function, increased progression free survival (PFS) and improved of patients’ quality of life [40]. In metastatic melanoma and renal cell carcinoma, autologous NK cells did not show any anti-tumor activity. Although NK cells persisted in PB they had lost NKG2D expression and needed to be re-activated with IL2 [41]. In 2008, Alici et al. also developed a technique to expand NK cells without the need of feeder cells. They managed to obtain a high number of activated NK cells starting from Peripheral Blood Mononuclear Cells (PBMC), by adding anti-CD3 antibody for the first five days and IL2 [43]. Afterwards, they compared different expansion systems by using flasks, cell culture bags and bioreactors and showed that bioreactors without the need of feeder cells obtained the best results [44]. Afterwards, they used this technique in a clinical trial with five refractory cancer patients who received donor-derived expanded NK cells after allo-SCT. In one patient (20%) with hepatocellular carcinoma partial responses were observed with markedly decreased serum alpha-fetoprotein levels [42]. Of interest, other type of feeder cells (Epstein-Barr virus immortalized lymphoblastoid B-cell lines: EBV-LCL) have also been tested to expand NK cells, also obtaining a high number of activated NK cells with efficient anti-tumor activity in mouse melanoma models [45]. More information about clinical studies infusing activated NK cells into patients has been addressed in other reviews [46]. 

All these studies demonstrated that: (1) there is a lack of expansion and persistence of NK cells in PB at long term, which is due to allo-reactive T cells eliminating NK cells [47,48]; (2) the negative immunosuppressive effect of T-reg cells might be improved with the use of IL2DT [26]; (3) NK cells might be a better choice for consolidation therapy rather than for refractory patients; (4) in non-myeloid refractory malignancies, NK cells do not achieve durable responses and the KIR-HLA-I ligand mismatch might not be always efficient. Moreover, after NK in vitro expansion, the KIR-HLA-I mismatch effect in some occasions can be bypassed, and the expression of NK receptors become homogenous because of the expansion. Therefore, in non-myeloid malignancies, activating receptors could be more relevant [49,50]; (5) different expansion techniques such as the aAPC K562-based system, NK cells derived from HPC and NK-92 cell line have been developed to overcome the limitation in obtaining large number of NK cells for the treatment of large tumor masses. These techniques allow obtaining a high number of NK cells ready to infuse off-the-shelf [29].

## 4. Chimeric Antigen Receptors (CAR) Modified NK Cells

Whereas CAR-T cell therapy has appeared in the last years as a revolutionary immunotherapy option for the treatment of hematological malignancies, CAR-modified NK cells is a field still under development. A CAR is a chimeric molecule composed of three regions: (1) an extracellular domain derived from the single chain variable fragment (scFV) of a monoclonal antibody (mAb), which redirects the specificity of T cells towards a specific target expressed in tumor cells without the need of antigen presentation; (2) a transmembrane domain; and (*3*) an intracellular domain derived from the ζ chain of the T cell receptor (TCR)/CD3 complex which activates the lytic pathway of T cells. Moreover, co-stimulatory signaling endodomains (CD28, 4-1BB or OX40) activate T cell proliferation after encounter of the target cell. The number of co-stimulatory domains can differ between the different CAR [51]. First clinical studies infusing CAR-T cells showed the efficacy of these cells in refractory patients [52]; consequently, an increasing number of clinical studies infusing CAR-T cells are currently being executed.

The intrinsic anti-tumor activity of NK cells added to the high number of activating receptors that initiate their cytotoxic activity would lead us to hypothesize that NK cells do not need a CAR. However, the negative clinical results infusing NK cells, especially in refractory patients, indicate that other options are needed. The addition of a CAR into NK cells might add an additional mechanism of tumor cell recognition, specifically useful in patients with down-regulation of the ligands required for activation of NK receptors. Furthermore, after recognizing the tumor cell, the CAR will induce NK cell proliferation increasing NK cell persistence in patients.

However, up to date, only preclinical studies using CAR-NK cells have been published. These include CAR-NK targeting CD19 and CD20 for B cell malignancies [53,54,55], CD5 for T cell malignancies [56], and CD138 and CS1 for MM [57,58]. In solid tumors, many preclinical studies have also been published targeting among others Her2, GD2 and EGFR for breast cancer, renal cell carcinoma, ovarian cancer, melanoma, neuroblastoma and glioblastoma [59,60,61,62]. Most of these studies have used NK-92 cells. However, other NK cell sources tested include NK cells previously obtained from HPCs [63], and NK cells from CB and expanded in vitro with aAPC K562-based systems [53]. More detailed information about current pre-clinical studies on-going with CAR-NK cells can be found in other reviews [64].

Some advances made in these studies indicate that the addition of IL15 into the CAR construct increases NK cell persistence in vivo [53]. Moreover, the inability of NK cells to traffic to tumor sites has been eliminated by the addition of C-X-X motif chemokine receptor 4 (CXCR4) in the CAR construct [65]. Clinical studies infusing CAR-NK are very scarce up to date and they are still recruiting patients. These studies target CD19 for B cell malignancies, CD33 for CD33+ AML and CD7 for leukemias and lymphomas. Most of these studies use either NK-92 cells or NK cells expanded in vitro with aAPC K562-based systems. They are summarized in Table 2.

## 5. CB Derived NK Cells (CB-NK): A Source of Highly Activated NK Cells Which Initiate a Transmissible Cytotoxicity

The aAPC K562-based system used to expand NK cells appears as one of the preferred techniques to expand NK cells in all published studies. These cells can have either membrane-bound IL15 or IL21, which are required for NK cell differentiation [66]. Therefore, this system, starting from mononuclear cells, allows obtaining a large number of differentiated NK cells. We have used this technique to expand NK cells from a CB unit. These NK cells are termed CB derived NK cells (CB-NK). At the end of the in vitro expansion, a large number of highly activated NK cells are obtained. Therefore, fully tested and HLA-typed NK cells are available off-the-shelf [50]. Interestingly, in physiological conditions, the CD56 bright NK cells are the immature population, with longer telomeres than CD56dim NK cells [67]. However, after in vitro expansion, CB-NK become a CD56bright cell population with a homogenous phenotype in terms of inhibitory KIRs and activating receptors, such as NKG2D and the NCR family of receptors [50]. Moreover, CB-NK show longer telomeres than freshly obtained NK cells from CB [68], and are highly activated in terms of both cytokine production and cytotoxicity [43,64].

We have studied the specific cytotoxicity of CB-NK against MM cells, which express HLA-I, and compared it against that of tumor cells with HLA-I down-regulation (K562 cells) [69]. We observed that CB-NK cytotoxicity differs for each type of tumor cell, in terms of NK cell receptors, cytotoxic molecules and types of cell death activated. Whereas NKG2D and NKP30 activating NK cell receptors do not have any impact in CB-NK cytotoxicity against K562, for MM cells, these receptors as well as NKG2D ligands have a significant role. Moreover, whereas Granzyme B is involved in CB-NK cytotoxicity against K562 cells mediating a Caspase-3 dependent cytotoxicity; in MM cells, Granzyme B does not impact the CB-NK cytotoxicity, which, in addition, is Caspase-3 independent. Moreover, this cytotoxicity against MM cells involves cathepsin release from lysosomes, which mediate a type of cell death termed “lysosomal cell death”. This dependence on cathepsins occurs only in MM cells and not in K562 cells [69], indicating the variety of cytotoxic mechanisms that NK cells can activate depending on each type of tumor cell.

Interestingly, when CB-NK and MM cells become in contact, both NKG2D and NKP30 receptors are transferred to MM cells. This transfer of NK cell receptors co-localizes with lipid structures. When Filipin-III—a lipid raft inhibitor—is added, this NK cell receptor transfer to MM cells decreases, suggesting a role of lipid metabolism in controlling the stability of these receptors in NK cells. NK cell activating receptors are continuously being recycled and degraded by endocytic pathway [70]. More specifically, the transfer of NKG2D and NKP30 co-localizing with proteins of the endocytic pathway (Rab1, Rab7 and Rab11) was confirmed [69]. Importantly, the transfer and degradation of these receptors in MM cells, might explain why cancer patients have NK cells that show with down-regulation of activating receptors [71,72].

**Figure 2:**
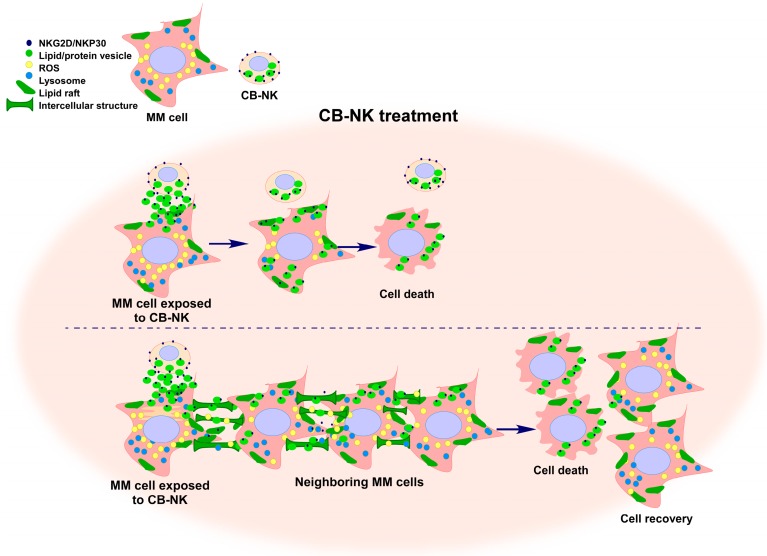
Transmissible cytotoxicity mediated by cord blood-derived NK cells (CB-NK) against multiple myeloma (MM) cells. When MM cells and CB-NK become in contact, NKG2D and NKP30 receptors are transferred into MM cells through lipid structures. A decrease in reactive oxygen species (ROS) and lysosome (Lys) levels is observed in MM cells exposed to CB-NK. When neighboring MM cells contact MM cells exposed to CB-NK, NK cell receptors are transferred into these neighboring MM cells, and Lys and ROS levels also decrease into neighboring MM cells. Consequently, a fraction of neighboring MM cells dies because of this CB-NK cytotoxicity. At the same time, ROS and Lys levels in MM cells exposed to CB-NK return to their original levels which could be a recovery of the initial damage caused by CB-NK.

Moreover, after CB-NK contact, MM cells become stressed and increase cell–cell communication between them. This increased cell–cell contact enables a secondary transfer of NKG2D and NKP30 NK cell receptors from MM cells exposed to CB-NK to neighboring MM cells non-exposed to CB-NK. Interestingly, this secondary transfer between MM cells of CB-NK proteins translates into a transmissible cytotoxicity mediated by MM cells, as initial MM cells exposed to CB-NK are able to transfer lipid-protein vesicles to neighboring MM cells non-exposed to CB-NK, causing cytotoxicity into a proportion of these neighboring MM cells (Figure 2) [69].

Remarkably, in addition to this novel transmissible cytotoxicity mechanism, we also observed that MM cells by establishing cell–cell communication among them; a diluent effect of the toxicity appears to be initiated. In particular, after CB-NK exposure, MM cells decrease their Reactive Oxygen Species (ROS) and Lysosome (Lys) levels. After contacting neighboring MM cells, this decrease in ROS and Lys is subsequently also observed in the neighboring MM cells, and surprisingly, the initial MM cells which contacted CB-NK manage to recover their original ROS and Lys levels (Figure 2) [69]. These events suggest a mechanism used by MM cells to recover from the damage caused by CB-NK. In this regard, increased cell–cell communication is a common event observed in cells after toxic events, such as cytotoxic drug administration, where damaged cells, by communicating with neighboring cells, propagate the damage across the cell population, and, therefore, they manage to recover [73,74,75]. Therefore, this transmissible effect mediated by tumor cells is a double edged-sword: on the one side, there is a transmissible cytotoxicity between MM cells; and, on the other side, it could be a tumor cell survival mechanism. This mechanism might decrease the final cytotoxic impact of NK cells, a finding that might have negative implications when using expanded NK cells as an immunotherapy option.

## 6. Tumor Cell Survival Mechanisms after Immunotherapy

Tumor cells develop many different types of immune evasion mechanisms that make them unrecognizable by NK cells. These mechanisms include down-regulation of MICA/B and ULBP1/3, the ligands of NKG2D [76,77]. Moreover, high levels of soluble ULBP [78], and B7-H6 [79], the ligands of NKG2D and NKP30, induce down-regulation of NK cell activating receptors, which correlate with metastases in patients [79]. In fact, it is common that cancer patients have hypo-responsive NK cells with down-regulation of NKG2D [71] and NKP30 [72]. Interestingly, high plasma level of soluble MICA/B, in Head and neck squamous cell carcinoma (HNSCC) patients correlates with NK cell inhibition, inability of NK cells to infiltrate HNSCC tumors, and disease progression, which was reverted in vitro after removal of soluble MICA/B from HNSCC patients’ plasma [80]. Moreover, the authors of this study tested successfully in rhesus monkeys an approach to remove soluble MICA from plasma by adsorption apheresis, a technique that could be used to improve cancer immunotherapy using NK cells [80]. A pro-inflammatory environment also contributes to NKG2D down-regulation, as release of the pro-inflammatory macrophage migration inhibitory factor (MIF) after inflammatory stimuli [81,82], contributes to NKG2D down-regulation and impaired NK cell cytotoxicity in cancer patients [83]. Interestingly, NKG2D down-regulation has been also observed after NK cell immunotherapy, leading to hypo-responsive NK cells [41]. This down-regulation of NK cell activating receptors associate with progressive disease [71], and also it has been suggested to occur due to chronic engagement of these receptors by their ligands expressed on tumor cells [72]. Another mechanism explaining this observed down-regulation of NK cell activating receptors could be due to the transfer of NK cell receptors between tumor cells after immune cell–tumor cell contact, as we have observed for CB-NK and MM cells. This transfer, occurring in part through lipid structures, could be avoided by previously inhibiting lipid metabolism in tumor cells. An interesting clinical approach would be to test approved and not toxic drugs involved in lipid metabolism inhibition, and to use them as co-adjuvants just before patients receive the immunotherapy treatment.

CAR-immunotherapy requires continuous engagement of the CAR with its target antigen on tumor cells, which unfortunately can lead to development of tumor cell immune evasion mechanisms, such as loss of expression of the target antigen. Actually, this loss of expression is one of the main problems after CAR-T cell therapy with concomitant patient relapse [84,85], which unfortunately will lead to 2nd and 3rd lines of CAR treatment targeting different antigens. Researchers have tried to solve this problem by using dual CARs, which target two antigens at the same time. However, even though a dual CAR might show superior activity [86], there is a risk for a relapse that will be resistant to two different antigens instead of one. Even though this phenomenon with CAR-NK has not been detected yet due to lack of studies, it is a possibility that needs to be analyzed. Moreover, after CAR-NK cell therapy, other types of tumor cell survival mechanisms have been detected, such as up-regulation by tumor cells of the immunosuppressive ligand HLA-G, which render CAR-NK unresponsive to tumor cells [87], and showing the variety of immune evasion mechanisms that tumor cells can develop after immunotherapy. The previously explained phenomenon of receptor transfer between cells, occurring also after CAR immunotherapy, could explain why some patients relapse. In particular, we observed that BCMA, a target antigen in MM cells and currently being tested for CAR immunotherapy [84], is transferred to CB-NK and to neighboring MM cells (Personal Communication: [88]). Avoiding this receptor transfer between cells as previously mentioned, by previously inhibiting lipid metabolism in tumor cells, could be also a strategy to improve CAR-NK immunotherapy.

The “immune checkpoints” which consist in pairs of receptors-ligands present in immune and tumor cells are also among the tumor cell survival mechanisms developed against NK cells. The interaction of these receptors in tumor cells with their ligands in immune cells inhibits the immune activity. Lymphocyte activation gene-3 (LAG-3), cytotoxic T lymphocyte antigen-4 (CTLA-4), programmed death-1 (PD-1) and T cell immunoglobulin and ITIM domain (TIGIT) are among the most studied immune checkpoints. Whereas immune checkpoints have been mainly studied in T cells, NK cells are also affected by these interactions. For instance, studies have shown that NK cells from MM patients express PD-1 whereas normal NK cells do not [89], and interestingly, monoclonal antibodies against PDL1 enhanced NK cell cytotoxicity in MM patients [90]. B7-H3, which is over-expressed in tumor cells, inhibits NK cell activity correlating with poor prognosis; and its inhibition improved NK cell activity controlling tumor growth [91]. PD-1 expression correlates with poor prognosis in digestive cancers, and its blockade promoted NK cell functions and suppressed tumor growth in hepatocellular carcinoma models [92]. TIM-3, which correlates with poor survival in melanoma, inhibits NK cell activity, and its blockade improved NK cell anti-melanoma activity in vitro [93]. Up-regulation of HLA-G in Ewing Sarcoma cells induced up-regulation of its receptor CD85j inhibiting NK cell activity [87]. Inhibiting all these interactions with monoclonal Antibodies, such as Ipilimumab (anti-CTLA-4), nivolumab (anti-PD-1) and others that are already being used in the clinic might also be useful in immunotherapy strategies infusing NK cells. These tumor cell survival mechanisms are summarized in Table 3.

## 7. Inflammatory Response: A Double-Edged Sword in Cancer

In addition to their anti-tumor role, NK cells are also well-known by their antimicrobial properties against fungal [94] and bacterial pathogens [95]. Granzymes and Granulysin are important mediators of this activity. In detail, Granzymes A and B are involved in the resolution of bacterial infection after *Escherichia coli (E. coli)*-induced peritonitis [96] and *Klebsiella*-induced pneumosepsis [97]. Granzyme M is involved in the antimicrobial NK cell activity against Listeria monocytogenes [98]; and Granulysin also show antimicrobial properties [99,100,101].

Unfortunately, the antimicrobial activity of these Granzymes correlates also with the production of pro-inflammatory mediators required to mount a general inflammatory response to eliminate pathogens. An inflammatory response prolonged and not resolved can lead to sepsis, but also to other inflammatory complications such as autoimmunity. Of interest, CD56 bright cells are associated to autoimmunity [102]. In more detail, Granzyme A and M are involved in the production of IL-1α, IL-1β, TNF, and IFNγ after bacterial stimuli, and also in the development of sepsis [103]. Granzyme M induces the production of MIP-1α after Listeria monocytogenes infection [98]. Granzyme K promotes a pro-inflammatory response with production of TNF-α, IL6 and monocyte chemotactic protein 1, is elevated sepsis, and also is released against activated T cells in multiple sclerosis [104,105,106]. Granzyme A is associated to autoimmune diseases such as arthritis [107]; and Granulysin acts as a chemoattractant activating monocytes to produce cytokines [108]. This pro-inflammatory activity of NK cells is also activated by the other cytotoxic mechanism that they can employ, the death receptors; as both, TRAIL receptors [109] and FASL [110] activate inflammation through TNF and NF-κB pathway [111,112].

Moreover, a prolonged and not resolved inflammatory response also leads to tumor cell proliferation. In fact, almost 2000 years ago, Galenus described the possibility that cancers evolved from inflammatory lesions. Therefore, the pro-inflammatory mediators released after NK cell immunotherapy in cancer patients is a factor that needs to be considered. On the one side, an acute inflammatory response of the immune system is needed to eliminate tumor cells. Actually, after CAR-T cell immunotherapy, the patients with the best clinical outcomes are the ones who develop the highest cytokine release syndrome (CRS) with the highest IL6 levels detected [84]. However, if this response is not resolved and prolonged, pro-inflammatory cytokines (IL1b, IL18, and IL6) might have a prejudicial effect activating inflammation and tissue damage [14,113], and, moreover, will lead to tumor cell proliferation. In fact, the same inflammatory molecules associated to NK cell activity, such as TNF-α, IL-1β and IL-6, are also elevated in advanced stage cancer patients correlating with risk of death [114]. Moreover, TNF and NF-κB pathway activated by TRAIL receptors and FASL, in addition to inducing tumor cell death, eventually will promote proliferation of tumor cells [111,112].

As previously mentioned, in vitro expanded NK cells used for immunotherapy, such as CB-NK, acquire a highly activated and inflammatory phenotype with bright CD56 expression, high production of Granzymes and other inflammatory mediators. We have performed proteomic studies to define the proteome released from CB-NK to MM cells and have observed a high variety of pro-inflammatory mediators including Granzyme A and Granulysin (Personal communication: [88]). Moreover, CAR-NK cells also require a process of in vitro expansion, indicating the pro-inflammatory potential of these cells. These pro-inflammatory properties acquired after in vitro expansion, suggest that a proper strategy for cancer immunotherapy, should also target inflammation in the long term to avoid the detrimental consequences of this response. Clinical protocols should include co-adjuvants to avoid chronic inflammation without eliminating the anti-tumor response. Future clinical studies performed with in vitro expanded NK cells will provide with more information about this issue.

## 8. Concluding Remarks

To summarize, clinical results infusing allogeneic NK cells have shown a clear benefit when infused as a consolidation therapy in AML patients. On the contrary, in refractory cancer patients with non-myeloid malignancies, NK cells have not yet been successful. NK cells do not proliferate in vivo and, moreover, many patients show down-regulation of NK cell activating receptors. In addition, tumor cells develop immune evasion mechanisms, including down-regulation of the ligands of NK cell activating receptors. Similarly, tumor cells are able to acquire NK cell activating receptors in part through lipid structures, which might explain the down-regulation of these receptors in NK cells. CAR-NK cell therapy will add an additional tumor cell recognition mechanism to compensate this imbalance in NK cell activating receptors, and, furthermore, will induce NK cell proliferation in vivo. In addition, a dilution of the NK cell damage performed by cell–cell communication between tumor cells has been observed, which might allow them to recover after NK cell treatment. Drugs inhibiting this tumor cell survival mechanism will improve clinical results using NK cells. Lastly, most NK cell sources infused into patients have been expanded in vitro acquiring high inflammatory properties. Therefore, clinical protocols will need to find the best protocol that target the inflammatory response after NK cell immunotherapy without impacting in the NK cell anti-tumor activity.

## Figures and Tables

**Table 1:** Clinical studies performed with NK (natural killer) cells as immunotherapy treatment.

*n* Patients Disease	NK Source	Treatment before NK Infusion	NK Activation	Detection of NK in PB	Median Number of Infused NK (×10^6^/Kg)	Graft vs. Host Disease	Outcome. Clinical Trial Number (Reference)
10. AML in CR (pediatric)	Haplo	Flu/Cy	IL2 post-infusion	Yes	29	No	All in remission at 964 days [24]
13. AML: 38.4% in AD, 15.3% MR, 46% CR	Haplo	Flu/Cy	IL2 post-infusion	Yes	2.74	No	AD: 20% achieved transient CR MR: 100% achieved CR CR: 50% DFS after 34, 32, 18 months. NCT00799799 [25]
16. AML in CR	Haplo	Flu/Cy	IL2 post-infusion	Yes	From 1.29 to 5.53	No	At 22.5 months: 56% DFS, 44% relapse. Higher NK cell number associated to higher DFS. NCT00799799 [28]
10. AML in CR	Allo NK derived from CD34+ HSPC from CB	Flu/Cy	IL15 and IL2	Yes (in 21%)	From 3 to 30	No	20% became MRD negative for 6 months [29]
57. Refractory AML (15 received IL2DT)	Haplo	Flu/Cy	IL2 post-infusion	Yes: in 10% of patients, and in 27% of patients receiving IL2DT)	26	No	CR: 53% (IL2DT) vs. 21% (no IL2DT) DFS: 33% (IL2DT) vs. 5% (no IL2DT) NCT00274846, NCT01106950 [26]
21. AML, MDS, CML	Haplo	Flu, Bu	IL2 pre and post-infusion	NA	From 0.22 to 8.32	No associated to NK	Survival associated with CD56+ cells delivered; 24% durable CR (no association to KIR-HLA mismatch). NCT00402558. NCT01390402 [27]
Refractory 6: AML, 2: MDS	Haplo	Flu/Cy	IL2 post-infusion	No	10.6	No	16% CR; 83% Disease progression. NCT00871689 [30]
29. Pediatric refractory AML Cohort 1: no prior allo-SCT (14) Cohort 2: relapsed after allo-SCT (15)	Haplo	Clo/Eto/Cy	IL2 post-infusion	Yes	From 3.5 to 103	No	Cohort 1: 71% response; 86% underwent allo-SCT; 36% DFS at 6 years. Cohort 2: 66.6% response and underwent allo-SCT; 27% DFS at 6 years. NCT00697671 and NCT00187096 [31]
7. High risk AML	Haplo	Flu/TBI	Tumor-primed NK cells with tumor lysate	Yes	3 doses: 1, 5, 10	No	At 6 months: 42.8% in CR remained in remission, 14% in PR achieved CR, 28% relapse, 14% died. At 1 year: 14% remained in CR. At 2 years: 85.7% died. Median OS: 400 days [32]
10. Relapsed MM	Haplo	Flu/Mel/Dx	IL2 pre and post infusion	Yes (until day 14)	1.7	No	50% CR or near CR, 20% PR, 10% SD and 20% PD [33]
17. Lymphoma (2), advanced solid tumors (15)	Allo	Non immunosuppressive regimen	IL2 (MG4101 method)	Yes	From 1 to 30 (1 and 3 doses)	No	Lymphoma: 50% SD, 50% PD Solid tumors: 47% SD, 53% PD. PFS in SD: 4 months. NCT01212341 [34]
5. Relapsed MM	Auto	Len, Bort	IL2, K562-mb15-41BBL cells	Yes	(7.5)x2	No	80% disease stabilization; 40–50% reduction in BM. NCT02481934 [35]
8. Relapsed MM	Auto/Haplo	Bort/Cy/Dx/Flu	K562-mb15-41BBL cells IL2 post-infusion	Yes (in 62%)	100	No	28% partial response [36]
12. Relapsed MM	CB	Len/Mel	K562-mb21-41BBL cells	Yes (in 50%)	4 doses: 5 , 10, 50 and 100	No	83% VGPR, 66% NCR; 33% relapse (at 21 months); 16% dead (at 21 months) [37]
6. Pediatric refractory solid tumors	Haplo	Flu/Bu/Thio/Mp	IL15	Yes	From 3 to 27	No	66% clinical response: 16% VGPR, 33% PR, 16% SD. At 310 days all patients died. NCT01337544 [38]
14. Ovarian 6. Breast	Haplo	Flu/Cy/TBI (in 7 pt)	IL2 pre and post-infusion	In 1 patient (no detection associated to T-reg presence)	21.6	No	Toxicity associated to TLS. NCT01105650 [39]
61.Hepatocellular carcinomoa Cryosurgery (26) Cryosurgery+NK (35)	Allo	Cryosurgery	K562-based system	NA	NA	No	Increased PFS: 9.1 vs. 7.6 months Increased Response rate: 60% vs. 46.1% Increased disease control rate: 85.7% vs. 69.2% [40]
7. Metastatic melanoma 1. Renal cell carcinoma	Auto	Flu/Cy	IL2	Yes	4.7	No	0% response. NCT00328861 [41]
5. CRC (1), .HC (1), RCC (2), CLL (1)	Allo	Ta/Mp (in 2 patients)	IL2 pre and post	Yes	From 1 to 50	No	20% PR [42]

Haplo: haploidentical; Allo: allogeneic; Allo-SCT: allogeneic stem cell transplantation; Flu: Fludarabine; Bu: Busulfan, ATG: Anti-Thymocyte Globulin; Ta: Tacrolimus, Mx: Methotrexate; Cy: Cyclophosphamide; Cs: Cyclosporine; Len: Lenalidomide; Bort: Bortezomib; Dex: Dexamethasone; Mel: Melphalan; Clo: Clofarabine, Eto: Etoposide; Thio: thiotepa; Mp: methylprednisolone; TBI: total body irradiation; TLS: tumor lysis syndrome; BM: bone marrow; AML: acute myeloid leukemia; MDS: myelodisplastic sindrome; CML: chronic Myeloid Leukemia; CLL: Chronic Lymphocytic Leukemia; NHL: Non-Hodgkin Lymphoma; MM: multiple myeloma; HC: Hepatocellular carcinoma; CRC: colorectal carcinoma; RCC: Renal cell carcinoma; CB: cord blood; HSPC: hematopoietic stem progenitor cells; VGPR: very good partial response; NCR: near complete response; PR: partial response; CR: complete remission; AD: active disease; MR: molecular relapse; SD: stable disease; PB: peripheral blood; PD: progressive disease; PFS: progression free survival; DFS: disease free survival; OS: overall survival; NA: information not provided in the study; IL2DT: IL2 diphtheria toxin fusion protein; NA: information not specified.

**Table 2:** Clinical studies on-going infusing CAR-NK cells in cancer patients.

NCT Number Institution	Type of NK/CAR-Co-Stimulatory Domains	Disease	Treatment/Doses
NCT02892695 PersonGen BioTherapeutics	NK-92 Anti-CD19-CD28, 4-1BB	Relapsed/refractory ALL, CLL, FL, BCL, DLBCL	NK before SCT
NCT02944162 PersonGen BioTherapeutics	NK-92 Anti-CD33-CD28, CD137	Relapsed/refractory CD33+ AML	NK on Days 0, 3 and 5
NCT02742727 PersonGen BioTherapeutics	NK92 Anti-CD7- CD28, 4-1BB	Relapsed/refractory CD7 positive leukemias and lymphomas	NK
NCT03056339 M.D.Anderson Cancer Center	CB-NK expanded with K562-mb21 Anti-CD19, 4-1BB, CD28, iCasp9, IL15	B-cell malignancies: ALL, CLL, NHL	Day-5 to -3: Flu, Cy, Mesna Day 0: NK AP1903 in case of CRS or GVHD
NCT02839954 PersonGen BioTherapeutics	NA	Relapsed/refractory Muc1 positive solid tumors	NA
NCT01974479 National University Health system, Singapore	Haploidentical NK expanded with K562-mb15-41BBL Anti-CD19, 4-1BB	Refractory ALL	NK
NCT00995137 St. Jude Children‘s Research Hospital	Haploidentical NK expanded with K562-mb15-41BBL Anti-CD19, 4-1BB	Refractory ALL	NK

ALL: acute lympoblastic leukemia; CLL: chronic lymphocytic leukemia; FL: follicular lymphoma; BCL: B cell lymphoma; MCL: mantle cell lymphoma; DLBCL: diffuse large cell lymphoma; AML: acute myeloid leukemia; NHL: non Hodgkin Lymphoma; Flu: fludarabine; Cy: cyclophosphamide; NA: information not specified

**Table 3:** Tumor cell survival mechanisms developed by tumor cells against NK cells.

Tumor Cell Survival Mechanism	Effect (Reference)
Down-regulation of MICA/B and ULBP1/3 (NKG2D ligands)	NK cell inhibition [76,77]
Increased levels of soluble ULBP (NKG2D ligand)	NKG2D down-regulation, NK cell inhibition [78]
Increased levels of soluble B7-H6 (NKP30 ligand)	NKP30 down-regulation, NK cell inhibition [79]
Increased levels of soluble MICA/B (NKG2D ligand)	NK cell inhibition [80]
Release of pro-inflammatory molecules (MIF)	NKG2D down-regulation [81,82]
Transfer of NKG2D and NKP30 to tumor cells	NKG2D and NKP30 down-regulation in NK cells [69]
Up-regulation of inhibitory HLA-G	CAR-NK unresponsive to tumor cells [87]
PDL1 over-expression	PD-1 interaction with subsequent NK cell inhibition [90]
B7-H3 over-expression	NK cell inhibition [91]
TIM-3 over-expression	NK cell inhibition [93]

NKG2D: also known as KLRK1 (killer cell lectin like receptor K1); NK: natural killer; NKP30: also known as NCR3 (natural cytotoxicity triggering receptor 3); CAR: chimeric antigen receptors; HLA: Human leukocyte antigen; MIF: migration inhibitory factor; PD-1: programmed cell death-1; PDL1: PD ligand 1; TIM-3: also known as PD-1.
